# Prof. N. S. Davis’ Letter

**Published:** 1857-08

**Authors:** N. S. Davis

**Affiliations:** Chicago


### Prof. N. S. Davis' Letter.

We insert below the honorable and manly reply of Prof. N. S. Davis to the Board of Health of Chicago, who had appointed him consulting physician to the new City Hospital. The rebuke Prof. D. thus administers to the official guardians of the public health of Chicago, might well be applied to those of a majority of our cities. We may remark that the assignment of certain wards of the Chicago Hospital to the medical care of Homœopathists is rendered most glaringly absurd when we remember that these globulists have just signally failed in sustaining a hospital under their own exclusive charge, and have abandoned it. But here is the letter:

CHICAGO, July 13, 1857.

*To the Hon. Board of Health of Chicago:*

GENTLEMEN: I had the honor on Saturday evening to receive from your secretary a communication informing me that I had been selected as one of the "consulting physicians of the *Allopathic* Medical Board," designed to take charge of a part of the new City Hospital.

Feeling a lively interest in whatever relates to the public health and welfare of our city, although my time is fully occupied, and I am already bestowing daily gratuitous services on one hospital, which admits an average of 500 patients annually, I would cheerfully assume the discharge of such additional duties as your proffered appointment would impose, had it been offered to me in an unobjectionable manner. But you ask me to become consulting physician to an "*Allopathic Medical Board.*" The word "allopathy," as applied to medicine, means a system of curing disease by *contraries;* that is, by setting up one disease in the system to eradicate or cure another. Although I have diligently studied and practiced medicine for more than twenty years, I must acknowledge that I know of no such system of medicine, and am profoundly ignorant of any class of men who pretend to practice any such system.

The word itself, as applied to the great body of physicians, conveys a libelous falsehood, which I will never sanction by accepting any appointment with which it is associated.

True and legitimate medicine acknowledges no *pathy*—no *ism*—no exclusive dogma of visionary enthusiasts; but it consists of the facts which have been gathered from every department of human knowledge by the accumulated experience, observation and research of centuries, and their application to the prevention and cure of disease.

But I have another objection to accepting the honor you offer. Whatever professional reputation (if any) I may have gained by twenty years of hard labor, would necessarily attach more or less to any public institution with which I might be connected, and this, too, without reference to its subdivisions or departments. Hence I could not, either consistently or conscientiously, allow my name to go before the public in connection with a hospital, a part of which is devoted to the treatment of the sick in accordance with an exclusive *pathy* or *pretended system*, which has already been fully tried and abandoned in the hospitals of almost every country in Europe. If your honorable body choose to make use of the poor and ignorant who may fall sick in our city (for it is such who will fill all public hospitals,) to test the merits of the various *pathys*, and *isms*, and humbugs of the day, you must do it without my assistance or sanction.

With sincere thanks for the honor which you intended to confer, I most respectfully decline to accept it. With much respect, yours, etc., N. S. DAVIS.

